# Sex differences in endocrine, metabolic and psychological disturbance in obese patients with OSA

**DOI:** 10.1186/s13293-025-00730-7

**Published:** 2025-07-01

**Authors:** Cheng Zi Qian, Zhang Li, Li Yi Ming, Han Teng, Su Lin Fan, Zhang Xiao Lei

**Affiliations:** 1 China-Japan Friendship Hospital (Institute of Clinical Medical Sciences), Chinese Academy of Medical Sciences & Peking Union Medical College; National Center for Respiratory Medicine; State Key Laboratory of Respiratory Health and Multimorbidity; National Clinical Research Center for Respiratory Diseases, Beijing, P.R. China; 2 Department of Respiratory and Critical Care Medicine, First Affiliated Hospital of Zhengzhou University, Zhengzhou, Henan, P.R. China; 3 Capital Medical University, Peking University Health Science Center, Beijing, P.R. China

**Keywords:** Obstructive sleep apnea, Sex difference, Endocrine, Metabolism, Psychological disturbance

## Abstract

**Background:**

Obstructive sleep apnea (OSA) is associated with increased risks of glucolipid metabolic disruption, endocrine disturbances and psychological distress. There is scarce research regarding the influence of sex on these associations. The current study aimed to evaluate the effects of sex on metabolic, endocrine and psychological changes in patients with OSA.

**Methods:**

One hundred sixty-four young adult women and one hundred sixty-two age-matched men with OSA completed polysomnography assessments, questionnaires (including the Epworth Sleepiness Scale [ESS], Self-Reported Anxiety Scale [SAS], Self-Rating Depression Scale [SDS], and 12-Item Short-Form Health Survey [SF-12]) and biochemical analyses for glucolipid metabolism and endocrine function, including the pituitary-adrenal (PA), pituitary thyroid (PT), and pituitary–gonadal (PG) axes.

**Results:**

Homeostasis model assessment of insulin resistance (HOMA-IR), thyroid hormone and midnight PA axis activity levels were greater in female patients with severe OSA compared to those with mild-to-moderate OSA, and these metabolic and endocrine changes were associated with nocturnal hypoxia only in female patients. Additionally, midnight cortisol was associated with HOMA-IR (independent of anthropometry and sleep disturbance parameters) in females (β = 0.545, P = 0.012, adjusted R^2^ = 0.217). ESS was higher for male patients with severe OSA compared to females with the same level of OSA (P = 0.003), and ESS was associated with nocturnal hypoxia in males (β = − 0.494, P = 0.001, adjusted R^2^ = 0.224). SAS was higher for female patients with severe OSA compared to males with the same level of disease (P = 0.001).

**Conclusions:**

The metabolic, endocrine and psychological consequences of OSA may differ across sexes. The associations of nocturnal hypoxia with glucose metabolic disturbance and the activation of the PA and PT axes were observed in females, whereas the association of nocturnal hypoxia with ESS was limited to males. This could indicate a distinct metabolic, endocrine and psychological phenotype for female patients with OSA, who may require different disease management strategies compared to males.

## Highlights

- The metabolic, endocrine and psychological consequences of obstructive sleep apnea (OSA) differ across sexes.
- Glucose metabolic disturbance, elevated thyroid hormone levels and the activation of the midnight pituitary-adrenal axis was associated with nocturnal hypoxia only in female patients.
- Midnight cortisol level was associated with homeostasis model assessment of insulin resistance (independent of anthropometry and sleep disturbance parameters) in female patients.
- The Epworth Sleepiness Scale score was higher for men with severe OSA compared to women with the same level of OSA, and it was associated with nocturnal hypoxia in male patients, whereas the Self-Reported Anxiety Scale score was higher for severe OSA patients than for mild-to-moderate OSA patients in females.

## Plain language summary

This study investigated the effects of sex on metabolic, endocrine and psychological changes in patients with obstructive sleep apnea (OSA). One hundred sixty-four young adult women and one hundred sixty-two age-matched men with OSA completed sleep monitoring assessments; questionnaire surveys regarding daytime sleepiness, anxiety, and depression; and biochemical tests for glucolipid metabolism and endocrine function (including hormones related to the adrenal gland, thyroid gland and gonads). We observed that women with severe OSA exhibited lower insulin sensitivity, elevated thyroid hormone levels, and greater activation of the adrenal axis at night than women with mild-to-moderate OSA. Interestingly, these metabolic and hormonal changes were linked to nighttime hypoxia only in women. Additionally, the activation of the adrenal axis at night was related to insulin sensitivity in women, which was independent of age, body weight and sleep disturbance. Daytime sleepiness was more sever for men with severe OSA compared to women with the same level of OSA, and daytime sleepiness was associated with nocturnal hypoxia in men, whereas anxiety was more severe for severe OSA compared to mild-to-moderate OSA in women. These findings suggest that the metabolic, endocrine and psychological changes associated with OSA may differ between men and women. Women are more prone to metabolic and hormonal disruptions caused by nocturnal hypoxia and are more vulnerable to anxiety, whereas men are more likely to experience daytime sleepiness as a key symptom.

## Background

Obstructive sleep apnea (OSA) is a prevalent disorder characterized by intermittent hypoxia and sleep fragmentation, which leads to and exacerbates cardiometabolic abnormalities, endocrine disturbances, cognitive impairments, and psychological distress [1]. OSA is more prevalent in men (with a prevalence of approximately 34%) than in women (with a prevalence of 17%), thereby highlighting a distinct sex disparity [2]. Sex differences in disease severity and symptom manifestations, as well as associated multiorgan impairments, have been reported in previous studies, which suggests that women may exhibit different OSA phenotypes than men [3–5]. Current disease management strategies, which are mainly based on clinical evidence solely obtained from male populations or negligent of sex disparity, may lead to underdiagnoses and inappropriate treatments of OSA in women. Recent studies have focused on this clinical gap and have strived to elucidate the underlying pathophysiological differences between the sexes, particularly in terms of hormonal regulation, which may influence both the manifestation and therapeutic responses of this disorder [6, 7].

The relationships between OSA and metabolic and endocrine diseases are bidirectional. Metabolic disorders (such as obesity, disturbances of glucose-lipid metabolism and metabolic syndrome) and endocrine disturbances (such as hypothyroidism and Cushing syndrome) are considered to be major risk factors for the development of OSA. Conversely, OSA may lead to and exacerbate these metabolic and endocrine disorders. Previous studies have reported different prevalences of metabolic alterations between male and female patients with OSA, which may indicate differences in the effects of sleep-disordered breathing between the sexes [3, 8]. Several factors have been postulated to contribute to or be critically involved in the genesis of dysmetabolic states in OSA, including sympathetic activation, inflammation, oxidative stress, and deregulation of the endocrine system [1, 9]. Among these factors, alterations in the hypothalamus-pituitary axis and the altered secretion of hormones from the peripheral endocrine glands may play important roles in the sex differences observed in the link between OSA and dysmetabolism. To date, very few studies have explored the influence of sex on the relationships between OSA and endocrine and metabolic abnormalities [10–12]. Moreover, OSA symptoms differ between females and males. Specifically, females are more likely to present with atypical symptoms, such as less snoring and greater frequencies of insomnia, mood disturbances, nightmares, fatigue and lack of energy [13]. However, the effects of sex on OSA symptoms (including daily alertness and psychological status) and overall quality of life have not been fully elucidated.

An understanding of the sex differences in the multisystem involvement of OSA patients is essential for the development of precision and personalised medicine. Thus, the main objective of this study was to investigate the sex differences in glucose-lipid metabolism, endocrine function (including the pituitary-adrenal [PA] axis, pituitary–gonadal [PG] axis and pituitary thyroid [PT] axis), and psychological status among patients with OSA. In addition, we aimed to investigate whether sleep breathing parameters are associated with metabolic, endocrine and psychological profiles, which are differentially modulated by OSA in males and females.

## Methods

### Participants

**Fig. 1:**
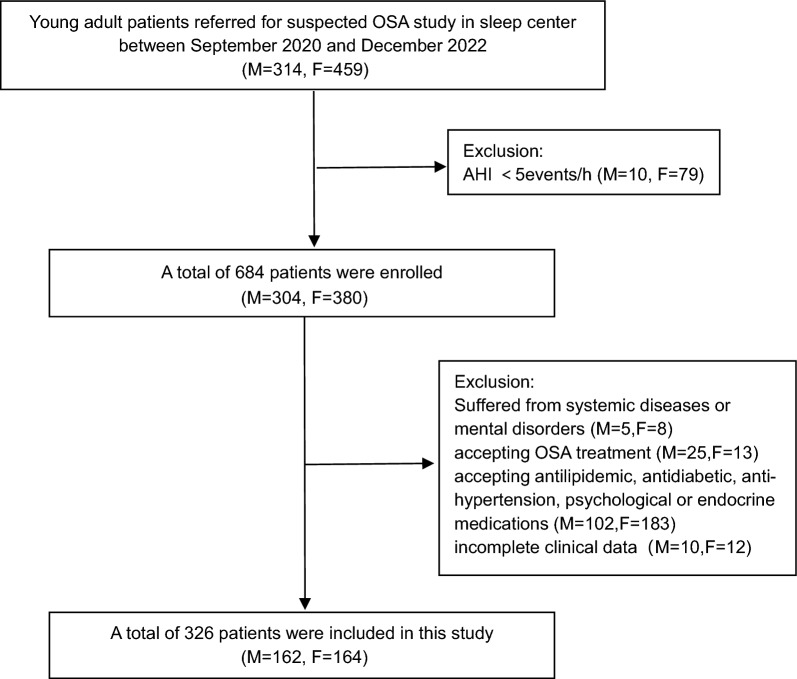
Flowchart of participants in this study. OSA = obstructive sleep apnea; AHI = apnea hypopnea index; M = male; F = female

This prospective, cross-sectional, observational study was conducted in the sleep center of the National Clinical Research Center for Respiratory Diseases, Beijing, China, from February 2023 to October 2024. Consecutive obese Han Chinese subjects aged 18–44 years with a body mass index (BMI) exceeding 30 kg/m^2^ were recruited from the Bariatric Clinic. Participants were excluded if they (1) had an apnea hypopnea index (AHI) less than 5 events/h; (2) accepted OSA treatment (such as noninvasive positive airway pressure, oral appliance, or upper airway surgery treatments); (3) suffered from systemic diseases (such as chronic pulmonary disease, congestive heart failure, chronic kidney disease or hepatic failure); (4) were receiving antidiabetic therapy, lipid-lowering treatment, antihypertension drugs or endocrine medications (such as thyroid, corticosteroid or sex hormone drugs); (5) had been diagnosed with clinically significant mental disorders (such as major depressive disorders, bipolar disorder, anxiety disorders, schizophrenia, obsessive–compulsive disorders, or posttraumatic stress disorder) or were receiving psychiatric medications; (6) were shift workers or night workers; or (7) had missing clinical data (Fig. 1). All the participants provided written informed consent, and the Institutional Review Board of China-Japan Friendship Hospital approved the protocol.

### Symptoms and anthropometry

Excessive daytime sleepiness (EDS) was assessed using the Epworth Sleepiness Scale (ESS) [14]. The Self-Rating Depression Scale (SDS) and Self-Rating Anxiety Scale (SAS) were used to evaluate depression and anxiety, respectively [15]. The 12-Item Short-Form Health Survey (SF-12) was used to evaluate quality of life [16]. These self-assessment questionnaires were administered before the performance of the sleep study. Anthropometric indices, including body mass index (BMI), waist circumference, and hip circumference, were also assessed.

### Sleep study

The individuals were asked to participate in a sleep study (Nox T3, Nox Medical Co. Iceland) during their routine sleep hours. All the data were scored by experienced PSG technologists based on the updated 2023 American Academy of Sleep Medicine criteria. OSA was defined as an AHI of ≥ 5 events/hour; furthermore, OSA severity was defined as follows: mild OSA (5 ≤ AHI < 15 events/hour), moderate OSA (15 ≤ AHI < 30 events/hour), and severe OSA (AHI ≥ 30 events/hour) [17].

### Biochemical analysis

Blood was collected from the participants on the following morning of the PSG after 10–12 h of fasting. Fasting plasma glucose (FPG), triglycerides, cholesterol, high-density lipoprotein (HDL) and low-density lipoprotein (LDL) were measured via the enzymatic colorimetric assay method (Beckman Coulter, Brea, USA). Fasting plasma insulin (FINS) was measured via electrochemiluminescence (Roche Diagnostics GmbH, Mannheim, Germany). Insulin resistance was evaluated using the Homeostasis Model Assessment of Insulin Resistance (HOMA-IR), which was calculated with the following formula: (FINS [µU/mL] × FPG [mmol/L])/22.5 [18]. Luteinizing hormone (LH), follicle stimulating hormone (FSH), estradiol, progesterone, testosterone and prolactin were measured via chemiluminescence immunoassay method (Beckman Coulter, Brea, USA). These sex hormone measurements were targeted at the follicular phase for females. Free and total thyroxine (FT4 and TT4, respectively), free and total triiodothyronine (FT3 and TT3, respectively), and thyroid stimulating hormone (TSH) were measured via the electrochemiluminescence immunoassay method (Roche Diagnostics GmbH, Mannheim, Germany). Adrenocorticotropic hormone (ACTH) and cortisol were collected at strict sampling times (0 AM and 8 AM) after patients had rested quietly for at least 30 min and were measured by a chemiluminescence assay method (Roche Diagnostics GmbH, Mannheim, Germany). The diurnal cortisol slope (DCS) was calculated using the following formula: DCS = ([cortisol]8am − [cortisol]0am)/16 [19]. All the measurements were performed according to the manufacturer’s instructions. Samples exceeding the detection limits were diluted or concentrated as required, with all results guaranteed to fall within the laboratory's committed reportable range.

### Statistical analysis

Patients were categorized by sex and severity of OSA. Data are presented as medians (interquartile ranges) or mean ± standard deviation, as appropriate. Normality was determined by the Kolmogorov–Smirnov test. The variables with a nonnormal distribution were standardized by using a logarithm scale (Log10) before further analyses were performed. Two-Way analysis of variance (ANOVA) was used to assess the main effect of sex or OSA and their interactive effects on questionnaire scores, PSG and biochemical parameters. Post hoc analyses were conducted to further explore group differences using the Tukey honest significant difference method (HSD) for pairwise comparisons. Linear regression models were constructed for ESS, psychological status, and metabolic and hormonal profiles using a stepwise procedure, with age, BMI, AHI, oxygen desaturation index (ODI), mean pulse oxygen saturation (MSpO_2_), lowest pulse oxygen saturation (LSpO_2_) and time with oxygen saturation < 90% (T90) considered as the independent variables. Multiple linear regression model assumptions were checked prior to the analysis. Data analysis was conducted using SPSS 27.0 (SPSS Inc., Chicago, Illinois, United States). The level of significance was set at P < 0.05.

## Results

### Anthropometry, questionnaire scores and sleep study

The current study included 326 patients with OSA (162 males and 164 females) for the final analysis. Two-way ANOVA revealed significant main effects of OSA (F = 25.176, df = 2, P < 0.001, η^2^ = 0.174) and sex (F = 10.705, df = 1, p = 0.001, η^2^ = 0.043) on BMI, but a non-significant OSA × sex interaction (F = 2.149, df = 2, P = 0.119, η^2^ = 0.018) (Table 1). Post hoc comparisons revealed that patients with moderate-to-severe OSA exhibited a higher BMI than patients with mild OSA for both sexes. SBP (F = 14.901, df = 1, P < 0.001, η^2^ = 0.059) and SAS (F = 6.948, df = 1, P = 0.009, η^2^ = 0.029) demonstrated only sex effects. Compared with male patients, female patients with mild-to-moderate OSA demonstrated lower SBP. The SAS score was greater in females with severe OSA than in females with mild OSA (P = 0.0036) and males with severe OSA (P = 0.001). Two-way ANOVA revealed a significant main effect of OSA (F = 3.949, df = 2, P = 0.021, η^2^ = 0.032), an non-significant main effect of sex (F = 1.424, df = 1, P = 0.234, η^2^ = 0.006), and a significant OSA × sex interaction on ESS (F = 3.914, df = 2, P = 0.021, η^2^ = 0.032). Post hoc comparisons revealed that males with severe OSA exhibited higher ESS scores compared to male patients with mild and moderate OSA (P < 0.001 and P = 0.006, respectively) and female patients with severe OSA (P = 0.003). No significant effects were observed for WHR, DBP, SF-12 or SDS. Female patients collectively demonstrated significantly lower AHI values compared to males (9.95 (7.60, 22.28) vs. 29.80 (11.85, 51.05), P < 0.001). Significant differences were observed in sleep breathing parameters among patients with different OSA severities (independent of sex), and no interaction between sex and OSA severity was observed. Multiple linear regression analysis (with questionnaire scores as the dependent variables and age, BMI, AHI, ODI, MSPO_2_, LSPO_2_, and T90 as the independent variables) revealed that ESS was associated with LSPO_2_ only in male patients (β = − 0.494, P = 0.001, adjusted R^2^ = 0.224) (Table 2). The SAS, SDS and SF-12 scores were not associated with body composition or sleep breathing parameters in either sex.

**Table 1:** Anthropometry, sleep study and questionnaire profiles

	Male			Female			P		
	Mild OSA (n = 48)	Moderate OSA (n = 36)	Severe OSA (n = 78)	Mild OSA (n = 103)	Moderate OSA (n = 33)	Severe OSA (n = 28)	OSA	SEX	OSA × SEX
Age, years	28.04 ± 6.25	28.47 ± 8.09	31.72 ± 7.79	31.69 ± 7.87	30.06 ± 6.89	34.18 ± 9.69	0.014	0.034	0.755
BMI, kg/m^2^	36.62 ± 4.17	45.21 ± 9.73^a^	44.88 ± 7.73^a^	35.93 ± 4.98	39.68 ± 6.05^a^*	41.57 ± 7.10^a^	< 0.001	0.001	0.119
WHR	0.96 (0.92, 0.99)	0.99 (0.93, 1.03)	0.97 (0.95, 1.03)	0.89 (0.85, 0.95)	0.90 (0.86, 0.98)	0.94 (0.90, 0.97)	0.826	0.273	0.536
SBP, mmHg	136.17 ± 12.11	141.94 ± 14.27	140.79 ± 15.85	129.30 ± 13.18*	129.69 ± 13.66*	135.35 ± 19.79	0.109	< 0.001	0.512
DBP, mmHg	85.00 ± 12.34	85.71 ± 11.77	87.15 ± 11.70	81.96 ± 8.90	82.97 ± 12.93	87.00 ± 15.44	0.186	0.229	0.802
ESS	4.00 (1.00, 6.00)	6.00 (2.50, 8.00)	7.00 (4.75, 11.50)^ab^	4.00 (2.00, 8.00)	5.00 (3.00,7.75)	6.00 (2.25, 9.00) *	0.021	0.234	0.021
SF-12	34.00 (32.25, 37.00)	33.12 ± 3.61	33.50 (30.00, 36.00)	32 (30, 34)	33 (31, 35)	33 (31, 35)	0.940	0.110	0.058
SAS	35.92 ± 6.21	35.06 ± 7.26	36.03 ± 5.88	36.41 ± 4.92	37.80 ± 5.97	39.78 ± 4.37^a^*	0.218	0.009	0.513
SDS	44.67 ± 6.87	41.93 ± 6.68	44.10 ± 8.49	44.59 ± 6.49	43.63 ± 5.84	44.68 ± 7.90	0.385	0.439	0.798
AHI, events/h	8.00 (4.35, 11.40)	20.90 (17.75, 25.35) ^a^	51.30 (41.20,79.10)^ab^	5.60 (3.40, 9.60)	20.95 (18.03, 24.13)^a^	53.85 (37.43, 74.65)^ab^	< 0.001	0.483	0.960
ODI, events/h	12.30 (8.53, 15.83)	22.80 (19.20, 34.95) ^a^	55.40 (38.80, 74.50)^ab^	7.40 (4.20, 12.30)	21.50 (16.93, 24.55)^a^	51.90 (40.20, 65.73)^ab^	< 0.001	0.097	0.985
AI, events/h	0.40 (0.05, 1.15)	0.70 (0.00, 2.45)	16.70 (3.10, 52.10)^ab^	0.30 (0.00, 0.60)	1.35 (0.53, 2.90)	16.15 (2.53, 45.13)^ab^	< 0.001	0.485	0.521
HI, events/h	6.75 (4.20, 11.23)	19.10 (16.40, 23.70) ^a^	31.90 (17.60, 42.10)^ab^	5.30 (3.00, 8.70)	18.35 (15.83, 21.78)^a^	34.35 (26.45, 44.68)^ab^	< 0.001	0.783	0.270
LSpO_2_, %	87.00 (84.50, 90.00)	81.00 (76.50, 84.00) ^a^	67 (60, 73)^ab^	86.00 (82.00, 89.00)	80.05 (74.50, 85.00)^a^	72.50 (62.25, 76.00)^ab^	< 0.001	0.762	0.371
MSpO_2_, %	95.00 (93.00, 96.00)	93.00 (92.00, 94.30)	89.40 (86.00, 92.45)^a^	95.40 (95.00, 96.00)	95.00 (93.30, 95.00)	91.80 (83.75, 94.25)^a^	< 0.001	0.417	0.961
T90, %	0.00 (0.00, 1.75)	5.00 (2.20, 12.00)	35.00 (17.00, 56.10)^a^	0.00 (0.00, 1.00)	4.00 (1.00, 9.75)	14.00 (8.40, 48.75)^a^	< 0.001	0.144	0.322

Data are shown as medians (interquartile ranges) or mean ± standard deviation

*Compared to the other sex with the same OSA severity

^a^Compared to mild subjects from the same sex group, ^b^compared to moderate subjects from the same sex group

OSA = Obstructive Sleep Apnea, BMI = Body Mass Index, WHR = Waist-to-hip ratio, SBP = Systolic blood pressure, DBP = Diastolic blood pressure, ESS = Epworth Sleepiness Scale, SF-12 = 12-Item Short-Form Health Survey, SAS = Self-Rating Anxiety Scale, SDS = Self-Rating Depression Scale, AHI = Apnea Hypopnea Index, ODI = Oxygen Desaturation Index, AI = Apnea Index, HI = Hypopnea Index, LSpO_2_ = Lowest pulse oxygen saturation, MSpO_2_ = Mean pulse oxygen saturation, T90 = Time of oxygen saturation below 90%

**Table 2:** Regression analysis of anthropometry and sleep breathing parameters with ESS, metabolism and endocrine profiles

	ESS		FBG		HOMA-IR		FT4		COR0am		ACTH0am		DCS	
	β	P	β	P	β	P	β	P	β	P	β	P	β	P
Female cohort														
Age	− 0.146*	0.047	− 0.049	0.285	− 0.183	0.116	− 0.004	0.168	0.048	0.508	0.201	0.168	− 0.007	0.108
BMI	− 0.124	0.228	− 0.038	0.556	0.282	0.088	0.000	0.923	0.004	0.966	0.200	0.305	0.005	0.425
WHR	− 0.802	0.160	− 0.084	0.814	0.500	0.581	0.017	0.421	− 0.151	0.771	− 0.558	0.588	0.011	0.841
AHI	0.172	0.138	− 0.035	0.635	− 0.204	0.268	− 0.007	0.133	0.251*	0.022	0.348	0.107	0.003	0.516
ODI	0.043	0.659	0.143	0.082	0.460*	0.028	0.009	0.061	0.058	0.665	− 0.405	0.089	− 0.002	0.621
MSpO_2_	0.103	0.711	0.077	0.661	0.305	0.492	0.014	0.188	-0.065	0.800	0.436	0.392	0.020	0.504
LSPO_2_	0.059	0.424	0.069	0.280	0.134	0.404	0.004	0.256	0.014	0.876	0.028	0.878	− 0.002	0.876
T90	− 0.110	0.382	− 0.046	0.335	− 0.101	0.403	0.275*	0.029	0.115*	< 0.001	0.318*	0.023	− 0.004*	0.046
Male cohort														
Age	0.008	0.951	0.088	0.151	0.081	0.667	-0.002	0.543	-0.094	0.310	0.278	0.359	-0.011	0.285
BMI	0.105	0.407	0.093*	0.042	− 0.022	0.905	0.002	0.487	0.122	0.201	0.803*	0.017	-0.013	0.206
WHR	16.71	0.120	1.422	0.778	− 4.752	0.763	0.019	0.949	11.99	0.129	12.99	0.613	1.659	0.057
AHI	− 0.113	0.198	0.055	0.193	0.090	0.490	− 0.001	0.754	− 0.038	0.573	− 0.321	0.155	0.002	0.748
ODI	0.075	0.154	− 0.027	0.290	0.043	0.585	− 0.001	0.527	− 0.008	0.826	0.023	0.850	− 0.009	0.484
MSpO_2_	0.017	0.971	− 0.287	0.197	− 0.174	0.801	0.026	0.053	0.061	0.858	1.762	0.139	− 0.012	0.210
LSPO_2_	− 0.494*	0.001	0.068	0.366	− 0.053	0.821	-0.004	0.360	− 0.117	0.323	-0.755	0.064	− 0.013	0.186
T90	0.029	0.703	− 0.061	0.099	− 0.068	0.545	0.034	0.544	0.004	0.070	0.194	0.297	− 0.087	0.668

BMI = Body Mass Index, WHR = Waist-to-hip ratio, AHI = Apnea Hypopnea Index, ODI = Oxygen Desaturation Index, MSpO_2_ = Mean SpO₂, LSpO_2_ = Lowest SpO₂, T90 = Time of SpO₂ below 90%, ESS = Epworth Sleepiness Scale, FBG = Fasting Blood Glucose, HOMA-IR = Homeostasis Model Assessment of Insulin Resistance, FT4 = Free Thyroxine, COR = Cortisol, ACTH = Adrenocorticotropic Hormone, DCS = Diurnal Cortisol Slope

*P < 0.05

### Glucose-lipid metabolic profiles

**Fig. 2:**
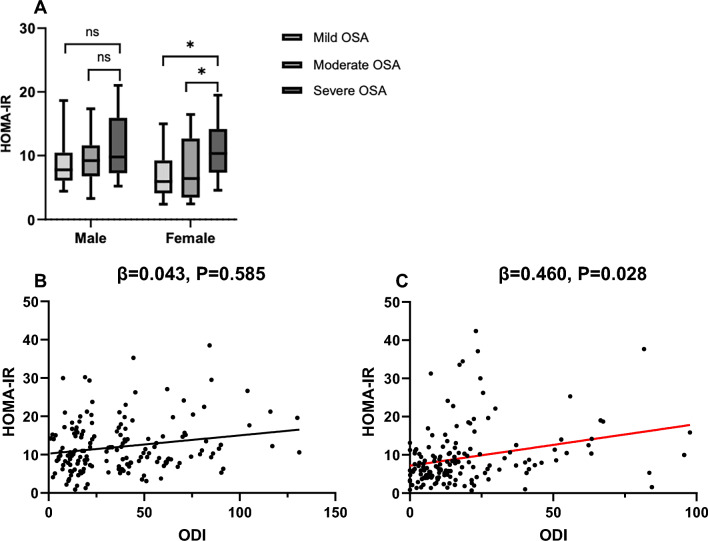
Analysis of glucose metabolism in patients with OSA. **A** Sex subgroup comparison of HOMA-IR in OSA patients with different severities of OSA, the notated significant differences represent post-hoc comparisons following Two-Way ANOVA testing. **B** Linear regression between HOMA-IR and ODI in males. **C** Linear regression between HOMA-IR and ODI in females. OSA = obstructive sleep apnea; HOMA-IR = Homeostasis Model Assessment of Insulin Resistance; ODI = oxygen desaturation index; *P < 0.05; ns = not significant

With respect to glucose-lipid metabolic profiles, two-way ANOVA demonstrated OSA effects on HOMA-IR (F = 4.722, df = 2, P = 0.010, η^2^ = 0.039), and no significant effects were observed for CHO, TRG, HDL-C, LDL-C or FBG (Table 3). Post hoc analysis showed that HOMA-IR were higher for females with severe OSA than for those with mild or moderate OSA (P = 0.001 and P = 0.005, respectively), however, this effect was not observed for males (Fig. 2A). Multiple linear regression analysis (with glucose-lipid metabolic profiles as the dependent variables and age, BMI, AHI, ODI, MSPO_2_, LSPO_2_, and T90 as the independent variables) revealed that HOMA-IR was independently associated with ODI in females (β = 0.460, P = 0.028, adjusted R^2^ = 0.224) but not in males (Fig. 2B and C). No body composition or sleep parameters were associated with lipid metabolic profiles in either sex.

**Table 3:** Glucose and lipid metabolic profiles

	Male			Female			P		
	Mild OSA (n = 48)	Moderate OSA (n = 36)	Severe OSA (n = 78)	Mild OSA (n = 103)	Moderate OSA (n = 33)	Severe OSA (n = 28)	OSA	SEX	OSA × SEX
Cholesterol, mmol/L	4.76 ± 0.91	4.70 ± 0.96	4.93 ± 0.95	4.9 ± 0 0.75	4.89 ± 0.96	4.98 ± 0.68	0.598	0.279	0.925
Triglycerides, mmol/L	1.66 (1.19,2.87)	1.54 (1.36, 1.93)	1.70 (1.22, 2.63)	1.36 (1.01, 1.93)	1.59 (1.31, 2.10)	1.49 (1.28, 2.35)	0.277	0.098	0.138
HDL, mmol/L	1.04 ± 0.22	0.92 ± 0.17	0.99 ± 0.18	1.14 ± 0.32	1.10 ± 0.26*	1.05 ± 0.21	0.157	0.004	0.489
LDL, mmol/L	2.92 ± 0.64	3.15 ± 0.62	3.32 ± 0.74	3.21 ± 0.68	3.22 ± 0.83	3.33 ± 0.58	0.110	0.205	0.463
FBG, mmol/L	5.93 (5.19, 9.59)	5.81 (5.57, 7.92)	6.31 (5.44, 7.59)	5.53 (5.01,6.48)	6.13 (5.38,7.83)	7.01 (5.47, 8.46)	0.807	0.415	0.341
HOMA-IR	8.75 (6.83, 11.47)	9.22 (6.44, 12.61)	9.48 (7.07, 16.06)	6.93 (5.09, 10.22)	7.53 (4.48,11.55)	10.41 (7.28, 14.59)^ab^	0.010	0.234	0.935

Data are presented as medians (interquartile ranges) or mean ± standard deviation

^a^Compared to mild subjects from the same sex group, ^b^compared to moderate subjects from the same sex group

HDL = High-Density Lipoprotein Cholesterol, LDL = Low-Density Lipoprotein Cholesterol, FBG = Fasting Blood Glucose, HOMA-IR = Homeostasis Model Assessment of Insulin Resistance

### Hormonal profiles

With respect to the PG axis hormones, two-way ANOVA demonstrated sex effects for FSH (F = 8.858, df = 1, P = 0.003, η^2^ = 0.036), LH (F = 11.989, df = 1, P = 0.001, η^2^ = 0.049), estrogen (F = 10.782, df = 1, P = 0.001, η^2^ = 0.045), and testosterone (F = 318.043, df = 1, P < 0.001, η^2^ = 0.576), with no significant effects being observed for prolactin or progesterone (Table 4). There were no significant differences observed in PG axis hormones between mild-to-moderate and severe patients of OSA in either sex. Multiple linear regression analysis revealed that no body composition indices or sleep breathing parameters were associated with PG axis hormones in either sex cohort.

**Table 4:** Hormone profiles

	Male			Female			P		
	Mild OSA (n = 48)	Moderate OSA (n = 36)	Severe OSA (n = 78)	Mild OSA (n = 103)	Moderate OSA (n = 33)	Severe OSA (n = 28)	OSA	SEX	OSA × SEX
Prolactin, mIU/L	274.57 (188.30, 377.01)	257.17 (201.77, 335.23)	229.96 (186.92, 274.40)	326.09 (221.36, 432.07)	260.56 (187.71, 366.91)	273.19 (219.07, 410.59)	0.180	0.471	0.057
FSH, mIU/mL	4.46 (3.51, 6.21)	3.62 (2.92, 6.52)	4.50 (3.08,5.64)	5.43 (4.06, 7.15)	6.04 (5.04, 7.58)*	5.86 (4.64, 8.32)*	0.190	0.003	0.236
LH, mIU/mL	4.34 (3.46, 6.56)	5.71 (3.63, 6.49)	4.02 (3.50, 5.45)	5.84 (3.66, 10.03)*	7.74 (4.58, 9.58)*	6.39 (3.51, 9.96)*	0.741	0.001	0.875
Estrogen, pmol/L	177.31 (125.21, 177.31)	167.57 (140.71, 207.76)	176.07 (134.46, 257.51)	241.21 (178.97, 364.42)*	246.32 (195.00, 424.43)*	227.06 (165.32, 323.11)*	0.798	0.001	0.773
Progesterone, nmol/L	0.81 (0.56, 1.12)	0.55 (0.45, 0.78)	0.62 (0.45,0.78)	0.80 (0.57, 1.94)	0.71 (0.57, 1.15)	0.61 (0.57, 0.88)	0.737	0.271	0.778
Testosterone, nmol/L	9.32 (8.33, 12.93)	8.75 (4.68, 10.98)	8.14 (6.75,11.17)	2.11 (1.62, 2.52)*	2.09 (1.71, 2.73) *	2.02 (1.13, 2.60)*	0.395	0.001	0.133
FT3, pg/mL	3.40 ± 0.36	3.42 ± 0.21	3.41 ± 0.41	3.30 ± 0.44	3.35 ± 0.38	3.44 ± 0.39	0.055	0.423	0.113
FT4, ng/dL	1.30 ± 0.17	1.31 ± 0.16	1.27 ± 0.17	1.22 ± 0.14*	1.25 ± 0.20	1.33 ± 0.16^ab^	0.629	0.311	0.025
TT3, ng/mL	1.17 ± 0.16	1.27 ± 0.21	1.22 ± 0.22	1.18 ± 0.20	1.25 ± 0.26	1.30 ± 0.20^a^	0.021	0.400	0.347
TT4, μg/dL	7.15 ± 1.33	7.78 ± 1.78	7.22 ± 1.52	7.46 ± 1.64	7.49 ± 1.33	8.25 ± 1.56^ab^*	0.205	0.117	0.047
TSH*,* μIU/mL	2.85 (2.40, 3.73)	2.74 (1.72, 3.68)	2.23 (1.58, 3.29)	2.78 (2.07, 3.90)	2.75 (1.97, 4.31)	2.72 (2.11, 3.78)	0.237	0.127	0.716
Cortisol (0AM), μg/dL	2.18 (1.25, 3.61)	2.22 (1.38, 4.60)	3.20 (1.26, 5.61)	1.50 (1.00, 2.96)	1.73 (1.05, 3.56)	5.08 (1.46, 7.90)^ab^*	0.002	0.963	0.001
Cortisol (8AM), μg/dL	13.93 (10.19, 15.51)	13.04 (9.47, 19.00)	12.71 (9.27, 16.41)	13.83 (9.66, 19.19)	12.12 (9.98, 15.00)	14.70 (10.70, 19.07)	0.360	0.880	0.408
DCS	0.74 (0.45, 0.88)	0.68 (0.35, 0.91)	0.55 (0.27, 0.94)	0.69 (0.39, 1.07)	0.67 (0.51, 0.80)	0.49 (0.25, 0.95)	0.048	0.931	0.359
ACTH (0AM), pg/mL	7.06 (4.38, 10.01)	6.69 (5.68, 12.10)	9.97 (5.23, 19.05)^a^#	6.98 (5.16, 9.65)	7.71 (5.24, 12.54)	9.82 (5.96, 19.94) ^ab^	0.001	0.675	0.158
ACTH (8AM), pg/mL	31.65 (21.82, 45.79)	29.11 (20.94, 40.83)	31.76 (19.91, 43.59)	29.73 (17.74, 43.90)	28.83 (13.56, 42.45)	28.03 (19.00, 35.00)	0.095	0.798	0.547

Data are shown as medians (interquartile ranges) or mean ± standard deviation

*Compared to the other sex with the same OSA severity

^a^Compared to mild subjects from the same sex group, ^b^compared to moderate subjects from the same sex group

Note: FSH = Follicle-Stimulating Hormone, LH = Luteinizing Hormone, FT3 = Free Triiodothyronine, FT4 = Free Thyroxine, TT3 = Total Triiodothyronine, TT4 = Total Thyroxine, TSH = Thyroid Stimulating Hormone, DCS = Diurnal Cortisol Slope, ACTH = Adrenocorticotropic Hormone

**Fig. 3:**
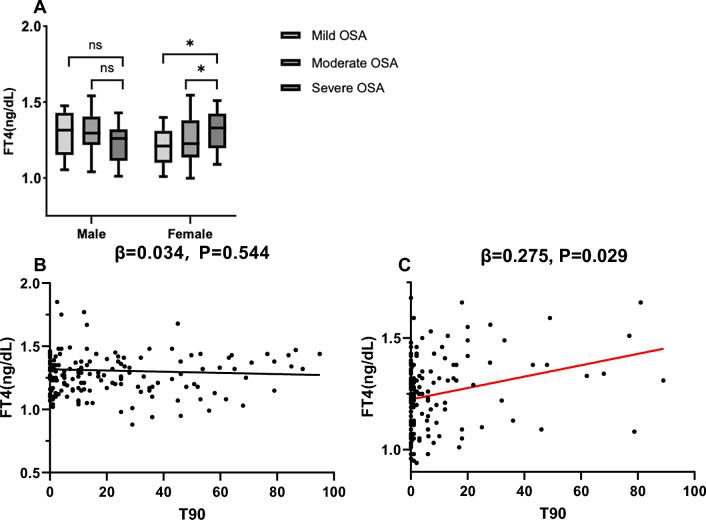
Analysis of the pituitary thyroid axis in patients with OSA. **A** Sex subgroup comparison of FT4 in OSA patients with different disease severities, the notated significant differences represent post-hoc comparisons following Two-Way ANOVA testing. **B** Linear regression between FT4 and T90 in males. **C** Linear regression between FT4 and T90 in females. FT4 = free thyroxine; OSA = obstructive sleep apnea; T90 = time of SpO_2_ below 90%; *P < 0.05; ns = not significant

For PT axis hormones, two-way ANOVA revealed a significant OSA × sex interactions for FT4 (F = 3.753, df = 2, P = 0.025, η^2^ = 0.031) and TT4 (F = 2.958, df = 2, P = 0.047, η^2^ = 0.026), whereas the main effects of neither sex nor OSA reached statistical significance. TT3 exhibited effects of OSA severity (F = 3.949, df = 2, P = 0.021, η^2^ = 0.032), and no significant effects were observed for FT3 or TSH. Post hoc comparisons revealed that FT4 and TT4 were higher in severe OSA patients than mild and moderate subjects in females (P = 0.005, P = 0.001, respectively), however, males did not demonstrate this effect (Fig. 3A). Multiple linear regression analysis (with PT axis hormones as the dependent variables and age, BMI, AHI, ODI, MSPO_2_, LSPO_2_, and T90 as the independent variables) revealed that FT4 was associated with T90 in the female cohort (β = 0.275, P = 0.029, adjusted R^2^ = 0.191), but not in males (Fig. 3B and C). No body composition or sleep breathing parameters were associated with PT axis hormones in the male cohort.

**Fig. 4:**
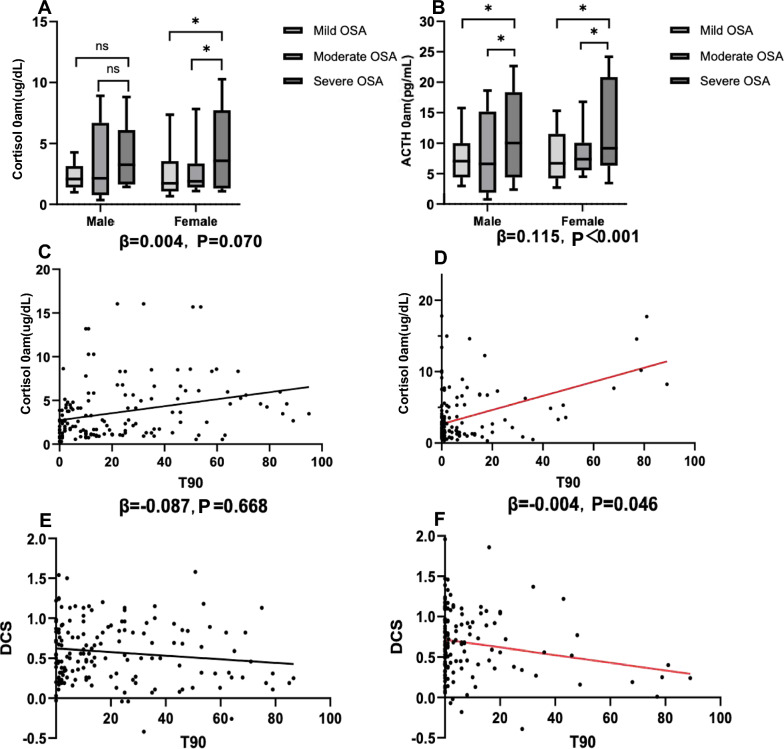
Analysis of the pituitary-adrenal axis in patients with OSA. **A** Sex subgroup comparison of cortisol measured at 0 AM in OSA patients with different severities of OSA, the notated significant differences represent post-hoc comparisons following Two-Way ANOVA testing. **B** Sex subgroup comparison of ACTH measured at 0 AM in OSA patients with different severities of OSA, the notated significant differences represent post-hoc comparisons following Two-Way ANOVA testing. **C** Linear regression between cortisol measured at 0 AM and T90 in males. **D** Linear regression between cortisol measured at 0 AM and T90 in females. **E** Linear regression between the DCS and T90 in males. **F** Linear regression between the DCS and T90 in females. OSA = obstructive sleep apnea; T90 = time of SpO_2_ below 90%; DCS = diurnal cortisol slope; *P < 0.05; ns = not significant

**Fig. 5:**
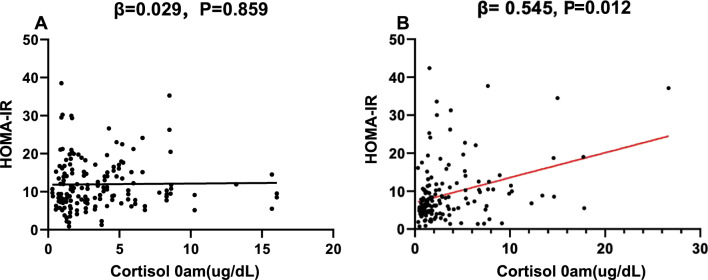
Regression analysis between HOMA-IR and cortisol measured at 0 AM in different sexes. **A** Linear regression between HOMA-IR and cortisol measured at 0 AM in males. **B** Linear regression between HOMA-IR and cortisol measured at 0 AM in females. HOMA-IR = Homeostasis Model Assessment of Insulin Resistance

Regarding the PA axis hormones, two-way ANOVA revealed significant main effects of OSA (F = 6.615, df = 2, P = 0.002, η^2^ = 0.056), non-significant main effects of sex (F = 0.002, df = 1, P = 0. 963, η^2^ = 0.001) on cortisol measured at 0 AM, and a significant OSA × sex interaction (F = 6.755, df = 2, P = 0.001, η^2^ = 0.057). ACTH at 0am (F = 7.464, df = 2, P = 0.001, η^2^ = 0.064) and DCS (F = 3.081, df = 2, P = 0.048, η^2^ = 0.027) demonstrated significant effects of OSA severity, and no significant effects were observed for cortisol or ACTH measured at 8 AM. Post hoc comparisons revealed that cortisol at 0am were higher for severe subjects compared with mild and moderate subjects in females (P = 0.002, P = 0.007, respectively), but not in males (Fig. 4A). ACTH at 0am were higher for severe subjects compared with mild and moderate subjects in both sexes (Fig. 4B). Moreover, the DCS tended to be flatter in patients with severe OSA than in those with mild and moderate OSA in both sexes; however, the differences were not statistically significant. There were no significant differences observed in cortisol or ACTH levels measured at 8 AM between patients of different OSA severities in either sex. Multiple linear regression analysis (with PA axis hormones as the dependent variables and age, BMI, AHI, ODI, MSPO_2_, LSPO_2_, and T90 as the independent variables) revealed that midnight cortisol and ACTH levels, as well as the DCS, were independently associated with T90 (β = 0.465, P < 0.001, adjusted R^2^ = 0.272; β = 0.318, P = 0.02, adjusted R^2^ = 0.201; β = − 0.004, P = 0.046, adjusted R^2^ = 0.178, respectively) in females, which was not observed among male patients (Fig. 4C–F). In addition, multiple lineal regression analysis with HOMA-IR as dependent variables and age, BMI, AHI, ODI, MSpO_2_, LSpO_2_, T90, midnight cortisol and DCS as independent variables, showed that midnight cortisol was independently associated with HOMA-IR in females (B = 0.545, P = 0.012, adjusted R^2^ = 0.217) (Fig. 5A and B).

## Discussion

In the current study, we demonstrated that HOMA-IR, thyroid hormones and midnight PA axis activity were greater in female patients with severe OSA than in those patients with mild-to-moderate OSA, and these metabolic and endocrine changes were associated with nocturnal hypoxia only in female patients. Additionally, midnight cortisol levels were associated with HOMA-IR (independent of anthropometry and sleep disturbance parameters) in females. Additionally, ESS was higher for men with severe OSA compared to women with the same level of OSA, and it was associated with nocturnal hypoxia in male patients, whereas SAS was greater for severe OSA patients than for mild-to-moderate OSA patients in females. Our findings suggest that peculiar sex differences exist in metabolism, endocrine, alertness and psychological statuses among patients with OSA and that nocturnal hypoxia could play an important role in regulating these alterations.

Several pivotal differences in sleep and sleep disorders have been recognized between males and females. In the current study, females demonstrated less severe OSA compared to males at comparable ages, which aligned with the findings of previous studies [20, 21]. The lower likelihood of premenopausal females developing OSA compared to males can be partly attributed to differences in the anatomy and respiratory physiology of the upper airways of females, which tend to shield females from obstructive events [7]. Specifically, females exhibit shorter airways, a more peripheral distribution of fat, and a lower airway collapsibility, which act as protective factors for OSA [22]. Previous studies have also revealed that females are more likely to report so-called atypical OSA symptoms, such as insomnia, fatigue, anxiety and depression; however, they do not exhibit typical symptoms, such as snoring and sleepiness. Our study revealed that the ESS score was higher for males and that the SAS score was higher for females, which was consistent with findings from previous studies. In addition, we observed higher nocturnal levels of cortisol in women. The HPA axis plays an important role in regulating sleep–wake homeostasis, and previous studies have reported that the activity of the HPA axis is associated with hyperarousal [23, 24]. As the major stress-responsive system, the HPA axis has also been implicated in mood regulation [25]. Whether these different manifestations of diurnal alertness and psychological status between the sexes in OSA patients are the result of elevated activation of the HPA axis in response to intermittent hypoxia or sleep structure fragmentation in females needs to be further clarified.

OSA was demonstrated to be associated with an increased risk of glucose-lipid metabolic disorders. To date, most research in this field has primarily focused on male patients, and most basic science studies have used male animals [26, 27]. Some studies have reported a higher prevalence of diabetes in male OSA patients and an association of OSA with diabetes only in males [28]. However, a long-term follow-up study demonstrated a markedly increased risk of new-onset diabetes in female patients, and OSA was observed to be an independent predictor of diabetes only in females [29]. In addition, an animal study of mice revealed that impaired glucose tolerance was evident in female intermittent hypoxia (IH) mice but not in males [30]. Our study revealed that HOMA-IR were greater for severe OSA patients than for mild-to-moderate OSA patients in females but not in males. Multivariate logistic regression analysis demonstrated that HOMA-IR was significantly associated with IH in female patients. Hormonal effects on pancreatic β cells and altered expression of metabolic genes were proposed to be important mechanisms of sex-dependent effects on glucose metabolism; however, these effects have rarely been investigated among patients with OSA and warrant further investigation. Currently, there is limited and controversial clinical research on the correlation between OSA and dyslipidemia, and few studies have explored the influence of sex on the association between OSA and dyslipidemia. A prior study reported that OSA is independently and positively associated with cholesterol, triglyceride and LDL and is negatively associated with HDL [31]. Silva WA et al. reported that male patients (but not female patients) with OSA were at increased risks of some measures of dyslipidemia [32]. In our study, we found OSA severity was not associated with dyslipidemia in either sex cohort. A possible explanation for this result may be that our patients were young adult obese individuals, and obesity may mask the effect of OSA on dyslipidemia. In addition, our female patients were evaluated prior to menopause, and estrogens play a major role in maintaining lipid metabolic homeostasis during this physiological cycle.

OSA is associated with several changes in pituitary function, among which the disruption of the HPA axis has received increasing attention. There is controversy regarding the link between the HPA axis and OSA according to the available literature. For example, some studies have demonstrated decreased activation of the HPA axis in OSA patients or have failed to reveal an association between OSA and activation of the HPA axis [33–35]. However, Kritikou et al. reported that three-month positive airway pressure treatment could revert increased cortisol levels to normal levels without any changes in body weight in patients with OSA [36]. In the current study, the midnight cortisol level was greater for patients with severe OSA than for those patients with mild-to-moderate OSA in females, and the levels of midnight cortisol and ACTH, as well as the DCS, were independently associated with nocturnal hypoxia. These changes were not observed among male patients. Diurnal cortisol secretion is a major aspect of PA axis regulation during adaptation to chronic stress. Intermittent hypoxia, arousal, and sleep fragmentation may lead to disturbances in the HPA axis. A previous study reported that females are more vulnerable to chronic stress [37]; however, sex differences in the activation of the HPA axis among patients with OSA have seldom been studied. We also observed that increased midnight serum cortisol levels were associated with increased HOMA-IR in female patients. Our understanding of the relationship between OSA and HPA axis activation, as well as its mediating effect on metabolism disturbances (especially regarding sex differences), remains quite limited.

As primary hormones, thyroid hormones exert profound effects on metabolism, development, and growth. Some research suggests that OSA may influence the HPT axis via intermittent hypoxia and sleep disturbances, whereas thyroid dysfunction can also increase the incidence of OSA by increasing upper airway collapsibility and suppressing hypoxic and hypercapnic ventilatory responses [38]. A recent meta-analysis reported that the prevalence rates of hypothyroidism, subclinical hypothyroidism and hyperthyroidism in OSA patients were 6%, 8% and 2%, respectively, and that there were no significant changes in thyroid hormone levels observed among OSA patients based on disease severity analysis [39]. In the current study, we demonstrated that thyroid hormone levels were higher in patients with severe OSA than in those with mild-to-moderate OSA among females; moreover, FT4 was independently associated with nocturnal hypoxia in the female cohort, which was not observed among male patients. Currently, the link between OSA and thyroid function has yet to be elucidated, especially regarding the effects of sex on HPT expression, which may be important for the necessity of routine screening of thyroid function in OSA patients.

Sleep is known to influence sex hormone cycles, and sleep disorders may have adverse effects on the HPG axis. Studies concerning the effects of OSA on serum sex hormone levels are relatively scarce. Some studies performed on males have reported that there was a negative correlation between testosterone levels and the severity of sleep apnea [40], whereas other researchers have reported that testosterone levels were similar between OSA patients and normal controls [41]. Females experience more fluctuations in hormonal changes during their lifespan. However, studies on the associations between sex hormones and OSA in females have mainly focused on hormone therapy during menopause. Moreover, associations between endogenous sex hormones and OSA in otherwise healthy childbearing-aged females have not yet been elucidated. Our results did not reveal associations between the HPG axis hormones and OSA severity in either sex cohort. The relationship between OSA and sex hormones is currently inconclusive, and well-designed and fully powered studies investigating the interventional effects of PAP or hormone therapy are needed.

Several limitations of this study should be noted. First, OSA is closely associated with ageing, and sex hormones play an important role in the incidence of OSA, especially among females; however, we recruited young adult patients (excluding postmenopausal females) from one medical Center. Thus, the specific characteristics of the current study population are worth noting, and our findings cannot be generalized to patients with different demographic characteristics. Second, due to its cross-sectional design, the results of our study did not establish a temporal relationship between OSA and metabolic and endocrine outcomes. Third, sex hormone dynamics throughout the menstrual cycle may influence insulin sensitivity, glucose metabolism, and psychological symptoms, although these effects may not occur in all females [42, 43]. Robust data concerning the menstrual cycle are lacking for the current study; therefore, our results need to be verified by further studies involving close monitoring of the phases of the menstrual cycle. Finally, obesity is quite prevalent among young adult patients with OSA, and the current analysis was obtained via multiple regression analysis controlling BMI and body composition parameters. Given the effect of adipose tissue on metabolic and endocrine parameters, additional studies including nonobese subjects are warranted to provide a better understanding of the impact of OSA on metabolism and endocrine parameters.

## Conclusions

In summary, our results demonstrate that the metabolic, endocrine and psychological consequences of OSA may differ between the sexes. The associations of insulin resistance, activation of the PA axis and activation of the PT axis with nocturnal hypoxia were limited to females, whereas the associations of nocturnal hypoxia with ESS were limited to males. Women are more vulnerable to anxiety, while men are more likely to experience daytime sleepiness. These distinct metabolic, endocrine and psychological characteristics across sexes underscore the necessity for an integrated approach for disease management that considers both disease severity and sex differences, in order to improve overall outcomes for patients with OSA.

## Abbreviations

ACTH: Adrenocorticotropic hormone
AHI: Apnea hypopnea index
AI: Apnea index
BMI: Body mass index
CHO: Cholesterol
DBP: Diastolic blood pressure
DCS: Diurnal cortisol slope
EDS: Excessive daytime sleepiness
ESS: Epworth sleepiness scale
FBG: Fasting blood glucose
FINS: Fasting plasma insulin
FPG: Fasting plasma glucose
FSH: Follicle-stimulating hormone
FT3: Free triiodothyronine
FT4: Free thyroxine
HDL: High-density lipoprotein
HDL-C: High-density lipoprotein cholesterol
HI: Hypopnea index
HOMA-IR: Homeostasis model assessment of insulin resistance
LDL: Low-density lipoprotein
LDL-C: Low-density lipoprotein cholesterol
LH: Luteinizing hormone
LSpO_2_: Lowest SpO₂
MSpO_2_: Mean SpO₂
ODI: Oxygen desaturation index
OSA: Obstructive sleep apnea
PA: Pituitary-adrenal
PG: Pituitary–gonadal
PT: Pituitary-thyroid
SAS: Self-rating anxiety scale
SBP: Systolic blood pressure
SDS: Self-rating depression scale
SF-12: 12-Item short-form health survey
T90: Time of SpO₂ below 90%
TRG: Triglycerides
TSH: Thyroid stimulating hormone
TT3: Total triiodothyronine
TT4: Total thyroxine
WHR: Waist-to-hip ratio

## Declarations

### Data availability

No datasets were generated or analysed during the current study.
